# Supplementary material for: Unveiling the Bioactive Potential of the Invasive Jellyfish Phyllorhiza punctata Through Integrative Transcriptomic and Proteomic Analyses
Source: Biomolecules. 2025 Aug 4;15(8):1121. doi: 10.3390/biom15081121 (PMC12383608; doi:10.3390/biom15081121)
Supplement: Supplementary file 1 [file biomolecules-15-01121-s001.zip › Suplementary Material/FileS3/PEPFOLD4/XP_065069205_13460/clusters.html]

 PEP-FOLD clusters


Download archive of all models  here   

|  |  |  |
| --- | --- | --- |
| # | file | sOPEP |
| # cluster 1 - Size: 28 |  |  |
|  | XP\_065069205\_13460-model1 | -25.9039 |
|  | XP\_065069205\_13460-model1.1 | -25.7588 |
|  | XP\_065069205\_13460-model1.2 | -25.5178 |
|  | XP\_065069205\_13460-model1.3 | -25.3128 |
|  | XP\_065069205\_13460-model1.4 | -24.99 |
|  | XP\_065069205\_13460-model1.5 | -24.6419 |
|  | XP\_065069205\_13460-model1.6 | -24.6368 |
|  | XP\_065069205\_13460-model1.7 | -24.5811 |
|  | XP\_065069205\_13460-model1.8 | -24.2958 |
|  | XP\_065069205\_13460-model1.9 | -24.0235 |
|  | XP\_065069205\_13460-model1.10 | -23.9395 |
|  | XP\_065069205\_13460-model1.11 | -23.88 |
|  | XP\_065069205\_13460-model1.12 | -23.6702 |
|  | XP\_065069205\_13460-model1.13 | -23.6483 |
|  | XP\_065069205\_13460-model1.14 | -23.5835 |
|  | XP\_065069205\_13460-model1.15 | -23.5687 |
|  | XP\_065069205\_13460-model1.16 | -23.5029 |
|  | XP\_065069205\_13460-model1.17 | -23.4272 |
|  | XP\_065069205\_13460-model1.18 | -23.3679 |
|  | XP\_065069205\_13460-model1.19 | -23.3546 |
|  | XP\_065069205\_13460-model1.20 | -23.0233 |
|  | XP\_065069205\_13460-model1.21 | -22.8946 |
|  | XP\_065069205\_13460-model1.22 | -22.8915 |
|  | XP\_065069205\_13460-model1.23 | -22.4987 |
|  | XP\_065069205\_13460-model1.24 | -22.4923 |
|  | XP\_065069205\_13460-model1.25 | -22.4867 |
|  | XP\_065069205\_13460-model1.26 | -22.3657 |
|  | XP\_065069205\_13460-model1.27 | -21.7003 |
| # cluster 2 - Size: 7 |  |  |
|  | XP\_065069205\_13460-model2 | -25.8052 |
|  | XP\_065069205\_13460-model2.1 | -23.3854 |
|  | XP\_065069205\_13460-model2.2 | -23.3298 |
|  | XP\_065069205\_13460-model2.3 | -22.9204 |
|  | XP\_065069205\_13460-model2.4 | -22.7948 |
|  | XP\_065069205\_13460-model2.5 | -22.6706 |
|  | XP\_065069205\_13460-model2.6 | -21.9686 |
| # cluster 3 - Size: 5 |  |  |
|  | XP\_065069205\_13460-model3 | -25.4873 |
|  | XP\_065069205\_13460-model3.1 | -25.469 |
|  | XP\_065069205\_13460-model3.2 | -25.3558 |
|  | XP\_065069205\_13460-model3.3 | -24.8573 |
|  | XP\_065069205\_13460-model3.4 | -23.1639 |
| # cluster 4 - Size: 7 |  |  |
|  | XP\_065069205\_13460-model4 | -25.473 |
|  | XP\_065069205\_13460-model4.1 | -24.6972 |
|  | XP\_065069205\_13460-model4.2 | -24.0079 |
|  | XP\_065069205\_13460-model4.3 | -23.67 |
|  | XP\_065069205\_13460-model4.4 | -23.5451 |
|  | XP\_065069205\_13460-model4.5 | -23.4639 |
|  | XP\_065069205\_13460-model4.6 | -23.2643 |
| # cluster 5 - Size: 1 |  |  |
|  | XP\_065069205\_13460-model5 | -25.3294 |
| # cluster 6 - Size: 12 |  |  |
|  | XP\_065069205\_13460-model6 | -25.0993 |
|  | XP\_065069205\_13460-model6.1 | -24.7481 |
|  | XP\_065069205\_13460-model6.2 | -24.7151 |
|  | XP\_065069205\_13460-model6.3 | -24.5565 |
|  | XP\_065069205\_13460-model6.4 | -24.5064 |
|  | XP\_065069205\_13460-model6.5 | -24.3333 |
|  | XP\_065069205\_13460-model6.6 | -24.0815 |
|  | XP\_065069205\_13460-model6.7 | -23.5887 |
|  | XP\_065069205\_13460-model6.8 | -23.4088 |
|  | XP\_065069205\_13460-model6.9 | -22.7694 |
|  | XP\_065069205\_13460-model6.10 | -22.7466 |
|  | XP\_065069205\_13460-model6.11 | -22.1262 |
| # cluster 7 - Size: 2 |  |  |
|  | XP\_065069205\_13460-model7 | -24.7167 |
|  | XP\_065069205\_13460-model7.1 | -21.6869 |
| # cluster 8 - Size: 2 |  |  |
|  | XP\_065069205\_13460-model8 | -24.6862 |
|  | XP\_065069205\_13460-model8.1 | -22.9625 |
| # cluster 9 - Size: 3 |  |  |
|  | XP\_065069205\_13460-model9 | -24.6667 |
|  | XP\_065069205\_13460-model9.1 | -24.572 |
|  | XP\_065069205\_13460-model9.2 | -23.062 |
| # cluster 10 - Size: 4 |  |  |
|  | XP\_065069205\_13460-model10 | -24.5747 |
|  | XP\_065069205\_13460-model10.1 | -24.3957 |
|  | XP\_065069205\_13460-model10.2 | -23.6218 |
|  | XP\_065069205\_13460-model10.3 | -23.0781 |
| # cluster 11 - Size: 3 |  |  |
|  | XP\_065069205\_13460-model11 | -24.5487 |
|  | XP\_065069205\_13460-model11.1 | -23.0461 |
|  | XP\_065069205\_13460-model11.2 | -22.7751 |
| # cluster 12 - Size: 5 |  |  |
|  | XP\_065069205\_13460-model12 | -24.4116 |
|  | XP\_065069205\_13460-model12.1 | -24.2816 |
|  | XP\_065069205\_13460-model12.2 | -24.0037 |
|  | XP\_065069205\_13460-model12.3 | -23.6041 |
|  | XP\_065069205\_13460-model12.4 | -23.5984 |
| # cluster 13 - Size: 10 |  |  |
|  | XP\_065069205\_13460-model13 | -24.2168 |
|  | XP\_065069205\_13460-model13.1 | -23.8383 |
|  | XP\_065069205\_13460-model13.2 | -23.7699 |
|  | XP\_065069205\_13460-model13.3 | -23.4853 |
|  | XP\_065069205\_13460-model13.4 | -23.4411 |
|  | XP\_065069205\_13460-model13.5 | -23.3006 |
|  | XP\_065069205\_13460-model13.6 | -23.2973 |
|  | XP\_065069205\_13460-model13.7 | -22.5846 |
|  | XP\_065069205\_13460-model13.8 | -22.5231 |
|  | XP\_065069205\_13460-model13.9 | -21.8206 |
| # cluster 14 - Size: 2 |  |  |
|  | XP\_065069205\_13460-model14 | -24.1925 |
|  | XP\_065069205\_13460-model14.1 | -24.1887 |
| # cluster 15 - Size: 2 |  |  |
|  | XP\_065069205\_13460-model15 | -24.0799 |
|  | XP\_065069205\_13460-model15.1 | -21.8218 |
| # cluster 16 - Size: 2 |  |  |
|  | XP\_065069205\_13460-model16 | -24.0045 |
|  | XP\_065069205\_13460-model16.1 | -23.8209 |
| # cluster 17 - Size: 1 |  |  |
|  | XP\_065069205\_13460-model17 | -23.3657 |
| # cluster 18 - Size: 2 |  |  |
|  | XP\_065069205\_13460-model18 | -23.0063 |
|  | XP\_065069205\_13460-model18.1 | -22.261 |
| # cluster 19 - Size: 2 |  |  |
|  | XP\_065069205\_13460-model19 | -22.9567 |
|  | XP\_065069205\_13460-model19.1 | -22.2519 |
|  |  |  |
